# Changes in Breast and Cervical Cancer Incidence by Stage at Diagnosis During the COVID‐19 Pandemic in Utah

**DOI:** 10.1002/cam4.70952

**Published:** 2025-05-19

**Authors:** Michelle Mumper, Leisha Nolen, Kimberly A. Herget, Rachel R. Codden, Marjorie E. Carter, Marie Nagata, Morgan M. Millar

**Affiliations:** ^1^ Minnesota Department of Health St. Paul Minnesota USA; ^2^ Utah Department of Health and Human Services Salt Lake City Utah USA; ^3^ Utah Cancer Registry University of Utah School of Medicine Salt Lake City Utah USA; ^4^ Division of Epidemiology Department of Internal Medicine, University of Utah School of Medicine Salt Lake City Utah USA

## Abstract

**Purpose:**

The COVID‐19 pandemic caused unprecedented disruptions in healthcare access, resulting in significant delays in breast and cervical cancer screening and diagnostic services. This study examined whether there were changes in the stage of diagnosis for breast and cervical cancers diagnosed among Utah women during the pandemic compared to years prior to the pandemic.

**Methods:**

Patients included adult females with a new breast or cervical cancer diagnosis reported to the Utah Cancer Registry, diagnosed from January 2020 to December 2021 (pandemic time period) or between January 2018 and December 2019 (pre‐COVID‐19). We calculated age‐adjusted incidence rates and incidence rate ratios (IRRs) with 95% confidence intervals (CI) to compare stage at diagnosis and sociodemographic factors between time periods.

**Results:**

A total of 308 cervical cancer cases and 8215 breast cancer cases were diagnosed throughout the duration of the study. Overall incidence of cervical cancer was higher during the pandemic, driven primarily by distant‐stage disease incidence, which was more than three times higher than before the pandemic (IRR, 3.11; 95% CI, 1.67–5.79). Non‐Hispanic (NH) White women were significantly more likely to be diagnosed with late‐stage cervical cancer (IRR, 1.60; 95% CI, 1.12‐2.30) during the pandemic compared to pre‐pandemic. Local‐stage breast cancer incidence decreased slightly during the pandemic compared to pre‐pandemic (IRR, 0.93; 95% CI, 0.88–0.99). Hispanic women saw a slight increase in late‐stage breast cancer incidence during the pandemic compared to before the pandemic (IRR, 1.31; 95% CI, 1.03–1.67).

**Conclusions:**

We saw a significant increase in the incidence of late‐stage cervical cancer during the pandemic compared with pre‐pandemic. Conversely, while local‐stage breast cancer incidence was slightly lower during COVID‐19 compared with pre‐COVID‐19, no difference was observed among all other stages. More time is needed to assess the full impact of COVID‐19 on breast and cervical cancer trends.

## Graphical abstract

This study examined whether there were changes in the stage of diagnosis for breast and cervical cancers diagnosed among Utah women during the pandemic (January 2020–December 2021) compared to years prior to the pandemic (January 2018–December 2019). We saw a significant increase in the incidence of late‐stage cervical cancer during the pandemic compared with pre‐pandemic. Conversely, while local‐stage breast cancer incidence was slightly lower during COVID‐19 compared with pre‐COVID‐19, no difference was observed among all other stages.

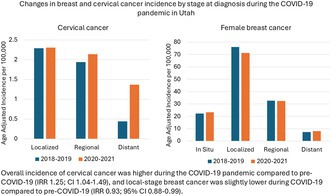

## 1 Introduction

According to the American Cancer Society, Utah is one of the lowest‐ranked U.S. states for adherence to breast and cervical cancer screening, ranked at 46 and 45, respectively [1]. Data from the Behavioral Risk Factors Surveillance System (BRFSS) Survey indicate that in 2020, breast cancer screening participation for Utah women ages 40+ was 63% compared to 69% nationwide [2], and cervical cancer screening participation for eligible women was 63% in Utah compared to 69% nationwide [3]. Screening rates in Utah have consistently fallen far below national averages for several years, making residents vulnerable to more adverse cancer outcomes. The declaration of the COVID‐19 national emergency in March 2020 presented additional barriers to breast and cervical cancer detection and management, including delays or even cancellations in screening and clinic appointments and postponed elective operations across the United States through initial stay‐at‐home orders [4, 5, 6, 7]. Screening for multiple types of cancer dropped sharply during this initial stay‐at‐home period across the country [8], with numerous women delaying breast and cervical cancer screenings [7, 9].

Any suspension of screening programs delays diagnosis and treatment of cancer, which contributes to advanced‐stage disease and lower survival probability [10, 11, 12]. The Utah Department of Health and Human Services (DHHS) Utah Cancer Control Program (UCCP), which provides free breast and cervical cancer screening services to low‐income women who are uninsured or underinsured, reported a sharp drop in cancer screening appointments during the implementation of the stay‐at‐home orders from March–May 2020. As a result, fewer underserved women were able to access screening services in 2020 compared with 2018 and 2019 [13]. However, while UCCP screening services were disrupted during the pandemic, overall mammography and Pap test screening rates in Utah for 2020 remained relatively stable. According to data collected by the Utah Behavioral Risk Factors Surveillance System (BRFSS), overall mammography and Pap test screening adherence did not differ significantly in Utah from 2019 to 2020 [2, 3]. Additionally, a recently published study looking at the Surveillance, Epidemiology, and End Results Program data found that, of the 22 central cancer registries analyzed, Utah had the smallest decline in incidence rates among all cancer sites while stay‐at‐home orders were in place [14].

While both breast and cervical cancer have screening tests, it is important to distinguish between the unique roles these tests play in the cancer detection and diagnosis process. The purpose of breast cancer screening is to detect breast cancer early, when treatment is most likely to be successful. However, the primary use of cervical cancer screening is not detection but prevention. That is, to identify and treat precancers before they become cervical cancer, which can take several years to develop if left untreated. That said, we may anticipate that the impact of the COVID‐19 pandemic on immediate incidence trends for breast and cervical cancer may vary.

In this study, we aimed to evaluate the impact of the COVID‐19 pandemic on the stage of breast and cervical cancer diagnoses among women in Utah using data obtained from the Utah Cancer Registry. Additionally, we sought to determine if changes in incidence patterns varied for underserved populations of women.

We assessed patient and clinical variables of female patients who were diagnosed with breast or cervical cancer in the years 2020–2021 when compared with a control cohort of diagnoses from 2018 to 2019. Due to the distinction in use of breast cancer screening for detection versus cervical cancer screening for prevention, we anticipated we may observe different outcomes for these two cancers in 2020–2021 as a result of the delayed and canceled screening appointments caused by the pandemic. We hypothesized that cervical cancer diagnoses would not change during the pandemic time period compared to the pre‐pandemic time period. Conversely, we hypothesized that breast cancer diagnoses would decrease during the pandemic time period compared to the pre‐pandemic.

## 2 Methods

### 2.1 Data

We performed a retrospective review of breast and cervical cancer diagnoses in Utah reported to the Utah Cancer Registry (UCR), a population‐based central registry that meets standards of completeness of case ascertainment as established by the U.S. Centers for Disease Control and Prevention's National Program of Cancer Registries (NPCR) and the National Cancer Institute's Surveillance, Epidemiology, and End Results (SEER) Program. Our sample included all female patients 18 years and older diagnosed with breast or cervical cancer from January 2020 through December 2021 (during the COVID‐19 pandemic, “pandemic time period”) and a comparison group of female patients aged 18 or older diagnosed January 2018 through December 2019 (“pre‐pandemic”). We included all stages of diagnosis for breast cancer, including in situ and invasive cases, and all invasive cases of cervical cancer. In situ cervical cancer is not a reportable diagnosis, and was therefore not included in this investigation. Patients diagnosed as unknown/unspecified stage were excluded. We limited the analysis to the first cancer a person was diagnosed with that met our inclusion criteria.

### 2.2 Variables

Cancer and sociodemographic characteristics were obtained from UCR records, based on their status at diagnosis. These included age, SEER Summary Stage 2018, race and ethnicity, urban/rural residence based on county‐level rural–urban continuum codes [15], census tract poverty category based on the American Community Survey tract‐level poverty data [16], and Utah local health district based on county at residence [17]. In addition, we considered the Utah Health Improvement Index (HII) score, which ranks Utah Small Areas from least to most socioeconomically disadvantaged based on nine indicators around economic, resource, and health factors [18]. “Utah Small Areas” refers to a set of geographic areas in Utah determined based on specific criteria, including population size, political boundaries of cities and towns, and economic similarity. For the purposes of this analysis, Utah Small Areas were categorized into one of five rankings according to the extent of their socioeconomic (SE) disadvantage as determined by their HII score: very low SE disadvantage, low SE disadvantage, average SE disadvantage, high SE disadvantage, and very high SE disadvantage. Clinical stage was determined based on how far the cancer had spread from its point of origin using the 2018 version of the National Cancer Institute Summary Staging Guide [19]. In response to a reviewer suggestion, we also evaluated breast cancer stage at diagnosis according to the American Joint Committee on Cancer (AJCC) staging system [20], using categories of 0, I, II, III, IV, and unknown. We maintained unknown AJCC‐stage cases in our analyses as they are a relatively large proportion of cases and coding instructions favor cancer registrars indicating unknown stage when uncertain, rather than trying to estimate a definitive stage. We were unable to analyze cervical cancer by AJCC stage as most cases did not have a known AJCC‐stage value in registry records. The large number of unknown values for AJCC stage for both breast and cervical cancer is also likely in part due the fact that this variable is not a required data item for central cancer registries to collect and submit to national standard setters, except for Commission on Cancer (CoC)‐accredited hospitals.

### 2.3 Analysis

Analyses of breast and cervical cancer diagnoses were performed separately using SAS (Version 9.4, SAS Institute Inc., Cary, NC). Patients with missing or unknown sociodemographic data were not excluded in order to accurately report and compare counts and incidence rates across select patient and clinical variables. We calculated counts and percentages of cancer cases for the COVID‐19 pandemic and pre‐pandemic time periods. We compared the distribution of cancer cases by sociodemographic factors between time periods using Pearson's chi‐squared test for independence, using the cutoff of *p* < 0.05 to determine statistical significance. We then computed age‐adjusted incidence rates and incidence rate ratios (IRRs) with 95% confidence intervals (CI) by stage at diagnosis to compare incidence rates (IRs) between the pre‐pandemic and pandemic time periods. IRRs with 95% CIs that did not contain 1.00 were considered statistically significant. Population estimates used to calculate incidence rates were obtained from Utah's Public Health Indicator Based Information System (IBIS), version 2021 [21].

Finally, we calculated age‐adjusted IRs and IRRs with 95% CIs by select sociodemographic characteristics and two categories of cancer stage at diagnosis: early‐stage cases (defined as in situ and localized for breast cancer and localized for cervical cancer) and late‐stage cases (defined as regional or distant disease). All sociodemographic variables that had reliable population estimates available to use in our analyses were included. IRRs comparing the pre‐pandemic and pandemic time periods with 95% CIs that did not include 1.00 were considered statistically significant.

## 3 Results

A total of 308 cervical cancer cases and 8215 breast cancer cases were diagnosed throughout the duration of the study: 133 cervical cancer cases and 4073 cases of breast cancer in the pre‐pandemic period and 175 cervical cancer cases and 4142 cases of breast cancer during the pandemic period. Demographic and stage characteristics summarized for the two time periods are shown in Table 1 and discussed in further detail for each cancer type below.

**TABLE 1:** Sociodemographics and stage of diagnosis of Utah cervical and breast cancer cases diagnosed in pre‐COVID and during‐COVID time periods.

Characteristic	Cervical cancer cases			Female breast cancer cases		
	2018–2019 (*n* = 133)	2020–2021 (*n* = 175)	2020–2021 vs 2018–2019	2018–2019 (*n* = 4073)	2020–2021 (*n* = 4142)	2020–2021 vs 2018–2019
	No. (%)	No. (%)	p*	No. (%)	No. (%)	p*
Cervical cancer age group			**0.039**			
< 40	51 (38.4)	45 (25.7)				
40–64	67 (50.4)	99 (56.6)				
65+	15 (11.3)	31 (17.7)				
Breast cancer age group						**0.024**
< 40				225 (5.5)	251 (6.1)	
40–49 years				636 (15.6)	719 (17.4)	
50–59 years				808 (19.8)	883 (21.3)	
60–69 years				1178 (28.9)	1111 (26.8)	
70–79 years				878 (21.6)	863 (20.8)	
80+ years				348 (8.5)	315 (7.6)	
Stage at diagnosis			**0.004**			0.121
In situ	N/A	N/A		659 (16.2)	710 (17.1)	
Localized	65 (48.9)	69 (39.4)		2259 (55.5)	2206 (53.3)	
Regional	55 (41.4)	63 (36.0)		946 (23.2)	978 (23.6)	
Distant	13 (9.8)	43 (24.6)		209 (5.1)	248 (6.0)	
AJCC stage at diagnosis***						0.301
0				582 (14.3)	594 (14.3)	
I				2409 (59.1)	2367 (57.1)	
II				396 (9.7)	447 (10.8)	
III				195 (4.8)	203 (4.9)	
IV				197 (4.8)	232 (5.6)	
Unknown				294 (7.2)	299 (7.2)	
Race/ethnicity			0.384			0.172
NH AIAN	**	**		17 (0.4)	25 (0.6)	
NH Asian	**	**		70 (1.7)	87 (2.1)	
NH Black	**	**		23 (0.6)	20 (0.5)	
Hispanic	26 (19.6)	36 (20.6)		390 (9.6)	447 (10.8)	
NH NHOPI	**	**		38 (0.9)	44 (1.1)	
NH White	94 (70.7)	129 (73.7)		3527 (86.6)	3505 (84.6)	
NH More than one race	**	**		**	**	
Unknown	**	**		**	**	
County Classification			0.840			0.687
Urban	116 (87.2)	154 (88.0)		3652 (89.7)	3725 (89.9)	
Rural	17 (12.8)	21 (12.0)		421 (10.3)	417 (10.1)	
Census tract poverty			0.643			0.193
0%–< 5% poverty	39 (29.3)	44 (25.1)		1520 (37.3)	1561 (37.7)	
5%–< 10% poverty	35 (26.3)	44 (25.1)		1174 (28.8)	1248 (30.1)	
10%–< 20% poverty	39 (29.3)	59 (33.7)		1108 (27.2)	1074 (25.9)	
20%–100% poverty	20 (15.0)	26 (14.9)		269 (6.6)	252 (6.1)	
Unknown	**	**		**	**	
Local health district			0.634			0.066
Bear river	**	**		226 (5.6)	183 (4.4)	
Central	**	**		105 (2.6)	98 (2.4)	
Davis	14 (10.5)	**		446 (11.0)	434 (10.5)	
Salt lake	58 (43.6)	77 (44.0)		1629 (40.0)	1696 (41.0)	
San Juan	**	**		12 (0.3)	**	
Southeast	**	**		54 (1.3)	36 (0.9)	
Southwest	**	17 (9.7)		385 (9.5)	425 (10.3)	
Summit	**	**		74 (1.8)	92 (2.2)	
Tooele	**	**		80 (2.0)	82 (2.0)	
Tricounty	**	**		64 (1.6)	51 (1.2)	
Utah	18 (13.5)	27 (15.4)		625 (15.3)	669 (16.2)	
Wasatch	**	**		31 (0.8)	46 (1.1)	
Weber Morgan	13 (9.8)	15 (8.6)		342 (8.4)	320 (7.7)	
Unknown	**	**		**	**	
Health improvement index category			0.394			**0.033**
Very low SE disadvantage	17 (12.8)	31 (17.7)		795 (19.5)	856 (20.7)	
Low SE disadvantage	31 (23.3)	42 (24.0)		1143 (28.1)	1233 (29.8)	
Average SE disadvantage	31 (23.3)	36 (20.6)		789 (19.4)	779 (18.8)	
High SE disadvantage	25 (18.8)	40 (22.9)		776 (19.1)	689 (16.6)	
Very High SE disadvantage	29 (21.8)	26 (14.9)		570 (14)	584 (14.1)	
Unknown	**	**		**	**	

*Note:* *p values comparing 2018–2019 and 2020–2021 data were calculated with Pearson's chi‐square test. Significant *p* values are highlighted in bold. ** The observed number of events is very small and not appropriate for publication. ***Not presented for cervical cancer cases due to a high proportion of missing data.

Abbreviations: AIAN, American Indian or Alaskan Native; NH, Non‐Hispanic; NHPI, Native Hawaiian or Pacific Islander.

### 3.1 Cervical Cancer

#### 3.1.1 Sociodemographic and Cancer Characteristics

There were 133 cervical cancers diagnosed in Utah in the pre‐pandemic period, and 175 diagnosed in the pandemic period. The mean age of diagnosis for cervical cancer patients in the pre‐pandemic group was 46.5 years compared to a mean age of 49.7 years among patients diagnosed in the pandemic time period. The age distribution of cervical cancer patients differed significantly between the pre‐pandemic and during‐pandemic time periods (*p* = 0.039, Table 1). Younger age diagnoses made up a smaller proportion in the pandemic period (25.7% younger than age 40) compared to the pre‐pandemic period (38.4% younger than age 40). Stage at diagnosis also differed significantly from pre to during the pandemic (*p* = 0.004). Before the pandemic, the distribution of stage at diagnosis consisted of 48.9% localized, 41.4% regional, and 9.8% distant compared to during the pandemic with 39.4% localized, 36% regional, and 24.6% distant. There were no statistically significant differences between the pre‐pandemic and pandemic periods in the distribution of cervical cancer patients by race and ethnicity, rurality, area‐level poverty, health district distribution, or HII. In terms of race and ethnicity, approximately 20% of cervical cancer patients in both time periods were Hispanic (19.6% pre‐pandemic and 20.6% pandemic period) and over 70% were non‐Hispanic White (70.7% pre‐pandemic and 73.7% pandemic period). A majority of cervical cancer cases were diagnosed in urban residents in both time periods (87.2% pre‐pandemic, 88.0% pandemic period), approximately 15% of cases resided in Census Tracts with a 20% or higher poverty rate in both periods, and slightly more resided in very high SE disadvantage areas in the pre‐pandemic period (21.8% compared to 14.9% during the pandemic, *p* = 0.394). Forty‐four percent of cervical cancer patients resided in the Salt Lake health district in both time periods.

#### 3.1.2 Incidence Rates

**FIGURE 1:**
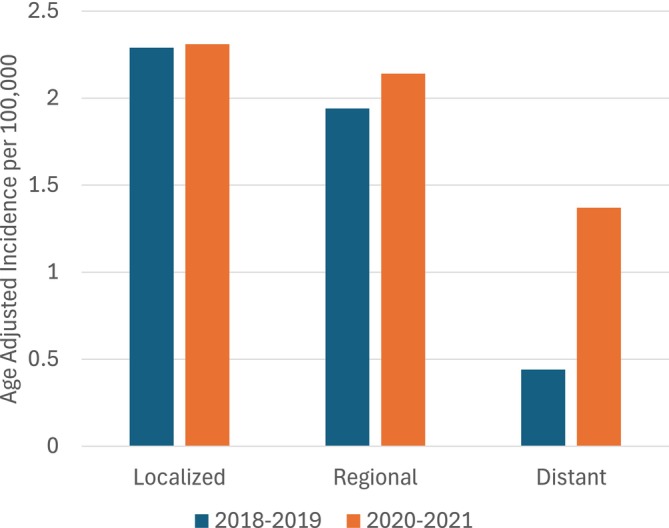
Age‐adjusted cervical cancer incidence in Utah, pre‐pandemic compared to pandemic period, by stage at diagnosis.

As shown in Figure 1, distant‐stage cervical cancer age‐adjusted incidence was more than three times higher during the pandemic than before the pandemic (IRR, 3.11; 95% CI, 1.67–5.79; Table 2). No significant differences were found between the age‐adjusted IRs of localized and regional disease by time period. However, for all invasive cervical cancers combined, the overall incidence was slightly higher during the pandemic compared to before the pandemic (IRR, 1.25; 95% CI, 1.04–1.49; Table 2).

**TABLE 2:** Age‐adjusted cervical cancer and female breast incidence rates* (IRs) and incidence rate ratios (IRRs) by stage at diagnosis, UCR, 2018–2021.

Variable		Cervical cancer			Female breast cancer		
		IR per 100,000		IRR (95% CI)	IR per 100,000		IRR (95% CI)
		2018–2019	2020–2021	2020–2021 vs. 2018–2019	2018–2019	2020–2021	2020–2021 vs. 2018–2019
Stage at diagnosis							
	In Situ	N/A	N/A	N/A	22.24	23.13	1.04 (0.94–1.16)
	Localized	2.29	2.31	1.01 (0.72–1.42)	76.13	71.16	**0.93 (0.88–0.99)**
	Regional	1.94	2.14	1.10 (0.77–1.58)	32.71	32.28	0.99 (0.90–1.08)
	Distant	0.44	1.37	**3.11 (1.67–5.79)**	7.16	8.10	1.13 (0.94–1.36)
	Total**	4.67	5.82	**1.25 (1.04–1.49)**	138.24	134.66	1.03 (0.98–1.07)
AJCC stage at diagnosis***							
	0				19.52	19.22	0.98 (0.88–1.10)
	I				81.39	76.43	**0.94 (0.89–0.99)**
	II				13.83	14.95	1.08 (0.94–1.24)
	III				6.72	7.13	1.06 (0.87–1.29)
	IV				6.76	8.10	1.20 (0.99–1.45)
	Unknown				10.01	10.37	1.04 (0.43–2.47)

*Note:* IRR that are significant are highlighted in bold. *Population estimates used to calculate incidence rates are provided by IBIS Version 2021. **Total incidence for cervical cancer includes localized, regional, and distant diagnoses. Total incidence for breast cancer includes in situ, localized, regional, and distant diagnoses. ***Not calculated for cervical cancer due to the high proportion of missing data.

Non‐Hispanic (NH) White women were significantly more likely to be diagnosed with late‐stage disease (IRR, 1.6; 95% CI, 1.12–2.3) during the pandemic time period compared to pre‐pandemic years (Table 3). A similar pattern was observed for Hispanic women, but this difference was not statistically significant (IRR, 1.63; 95% CI, 0.81–3.27). Additionally, late‐stage cervical cancer incidence was more than three times higher among rural residents and slightly higher among urban residents during the pandemic than before (rural IRR, 3.15; 95% CI, 1.04–9.58; urban IRR, 1.39; 95% CI, 1.01–1.91). Moreover, high SE disadvantaged Utah Small Areas had higher late‐stage incidence during the pandemic time period than before (IRR, 2.53; 95% CI, 1.24–5.14). No other significant differences were observed among the other sociodemographic characteristics by time period and early‐ or late‐stage diagnoses for cervical cancer.

**TABLE 3:** Age‐adjusted cervical cancer incidence rates* (IRs) and incidence rate ratios (IRRs) for early‐ and late‐stage diagnoses** by select sociodemographic characteristics, Utah, 2018–2021.

Variable	Early‐stage cervical cancer diagnoses			Late‐stage cervical cancer diagnoses		
	IR per 100,000		IRR (95% CI)	IR per 100,000		IRR (95% CI)
	2018–2019	2020–2021	2020‐2021 vs 2018‐2019	2018–2019	2020–2021	2018–2019 vs 2020–2021
Race/Ethnicity						
Hispanic, all races	3.32	4.20	1.26 (0.61–2.63)	3.53	5.74	1.63 (0.81–3.27)
NH White	14.50	14.04	0.97 (0.66–1.42)	1.97	3.16	**1.60 (1.12–2.30)**
County classification						
Urban	2.32	2.65	1.14 (0.79–1.65)	2.93	4.06	**1.39 (1.01–1.91)**
Rural	2.31	0.97	0.42 (0.17–1.05)	0.66	2.06	**3.15 (1.04–9.58)**
Health improvement index category						
Very low SE disadvantage	2.07	2.71	1.31 (0.61–2.83)	1.16	2.48	2.13 (0.83–5.50)
Low SE disadvantage	1.77	1.67	0.94 (0.44–2.04)	2.53	3.92	1.55 (0.86–2.80)
Average SE disadvantage	1.98	2.11	1.06 (0.49‐2.30)	3.08	3.28	1.06 (0.58‐1.97)
High SE disadvantage	2.84	2.77	0.98 (0.47‐2.03)	1.87	4.73	**2.53 (1.24‐5.14)**
Very High SE disadvantage	3.51	2.58	0.73 (0.34‐1.60)	3.49	3.66	1.05 (0.51‐2.17)

*Note:* IRR that are significant are highlighted in bold. *Population estimates used to calculate incidence rates are provided by IBIS Version 2021. **Early‐stage is defined as localized disease; late‐stage is defined as regional or distant disease.

Abbreviation: NH, Non‐Hispanic.

### 3.2 Breast Cancer

#### 3.2.1 Sociodemographic and Cancer Characteristics

There were 4,073 breast cancer cases diagnosed pre‐pandemic and 4,142 diagnosed during the pandemic period. The mean age of diagnosis for breast cancer patients in the pre‐pandemic group was 61.67 years compared to a mean age of 60.67 years among patients diagnosed in the pandemic time period. The distribution of age at diagnosis for breast cancer patients varied significantly between the pre‐pandemic and pandemic time periods (*p* = 0.024, Table 1), with age at diagnosis trending slightly younger in the pandemic period. Stage at diagnosis did not differ significantly (*p* = 0.121), and neither did race and ethnicity, county classification, poverty, and local health district (LHD). In both time periods, just over half of the cases were diagnosed at local‐stage disease (55.5% pre‐pandemic, 53.3% during the pandemic period). AJCC stage was also similar across both time periods, with 57%–59% stage I and 10%–11% stage II (*p* = 0.301). In both time periods, Hispanic or Latina patients comprised approximately 10% of breast cancer cases (9.6% 2018–2019, 10.8% 2020–2021) and non‐Hispanic White patients comprised the majority of cases (86.6% pre‐pandemic, 84.6% pandemic). Approximately 10% of cases were from rural counties in both time periods, and 37% of cases were diagnosed in Census Tracts with less than 5% poverty in both time periods. Forty to 41% of patients diagnosed with breast cancer resided in Salt Lake health district in both time periods. The distribution of cases across Utah Small Areas ranked by SE disadvantage varied significantly between the pandemic group compared to the pre‐pandemic group, shifting toward Utah Small Areas experiencing lower SE disadvantage during the pandemic (*p* = 0.033).

#### 3.2.2 Incidence Rates

**FIGURE 2:**
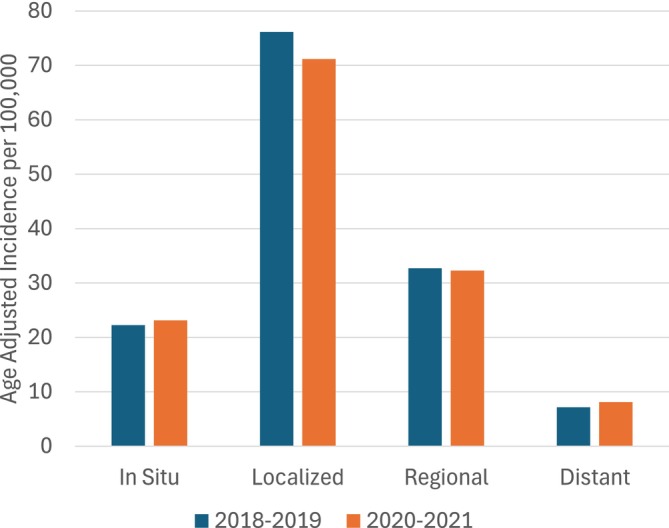
Age‐adjusted breast cancer incidence in Utah, pre‐pandemic compared to pandemic period, by stage at diagnosis.

The age‐adjusted incidence rate for breast cancer of any stage was 134.66 per 100,000 women during the pandemic period and 138.24 per 100,000 during the pre‐pandemic period. There was no statistically significant change in overall breast cancer incidence across the two time periods (IRR, 1.03; 95% CI, 0.98–1.07). Age‐adjusted incidence rates (IRs) and incidence rate ratios (IRRs) by stage at diagnosis among breast cancer cases are summarized in Table 2 and Figure 2. The IRRs showed a minor decline in cases of localized breast cancer during the pandemic compared with the pre‐pandemic time period (IRR, 0.93; 95% CI, 0.88–0.99). No other significant changes in breast cancer rates by stage at diagnosis were observed between the two time periods. Similarly, for AJCC stage at diagnosis, there was a significant decrease in the incidence of stage I disease (IRR, 0.94; 95% CI, 0.89–0.99) but no other changes across time periods were statistically significant. We also evaluated the change in the incidence of any late‐stage diagnosis (stages II–IV combined). Overall, late‐stage diagnoses increased slightly, but the difference was not statistically significant (IRR, 1.06; 95% CI: 0.97–1.17).

Table 4 presents age‐adjusted IRs and IRRs by select sociodemographic characteristics for early‐ and late‐stage cases. Incidence of late‐stage breast cancer among the Hispanic population exhibited a slight increase during the pandemic compared to before the pandemic (IRR, 1.31; 95% CI, 1.03–1.67, Table 4), but similar patterns were not observed for other race or ethnic groups. Additionally, during the COVID‐19 pandemic, urban areas exhibited slightly lower early‐stage breast cancer incidence (IRR, 0.85; 95% CI, 0.80–0.90) and late‐stage breast cancer incidence (IRR, 0.81; 95% CI, 0.74–0.88) compared to the pre‐pandemic time period (Table 4). No significant differences in early‐ or late‐stage breast cancer diagnoses were found among the other sociodemographic characteristics by time period.

**TABLE 4:** Age‐adjusted female breast cancer incidence rates* (IRs) and incidence rate ratios (IRRs) for early‐ and late‐stage diagnoses** by select sociodemographic characteristics, Utah, 2018–2021.

Variable		Early‐stage breast cancer diagnoses			Late‐stage breast cancer cases		
		IR per 100,000		IRR (95% CI)	IR per 100,000		IRR (95% CI)
		2018–2019	2020–2021	2020–2021 vs. 2018–2019	2018–2019	2020–2021	2020–2021 vs. 2018–2019
Race/ethnicity							
	NH AIAN	35.24	56.69	1.61 (0.71–3.64)	32.32	28.53	0.88 (0.34–2.29)
	NH Asian	55.85	74.85	1.34 (0.91–1.96)	32.50	25.80	0.79 (0.45–1.40)
	NH Black	77.56	72.62	0.94 (0.46–1.92)	42.90	21.18	0.49 (0.16–1.51)
	Hispanic	103.24	96.67	0.94 (0.79–1.10)	39.74	52.22	**1.31 (1.03–1.67)**
	NH NHPI	94.04	129.49	1.38 (0.78–2.43)	85.71	61.98	0.72 (0.36–1.43)
	NH White	99.91	96.09	0.96 (0.91–1.02)	40.35	39.64	0.98 (0.90–1.07)
County classification							
	Urban	135.94	115.44	**0.85 (0.80–0.90)**	60.74	49.07	**0.81 (0.74–0.88)**
	Rural	39.30	40.51	1.03 (0.88–1.21)	19.54	16.56	0.85 (0.66–1.09)
Health Improvement Index Category							
	Very low SE disadvantage	111.63	109.20	0.98 (0.80–1.20)	39.14	42.02	1.07 (0.89–1.30)
	Low SE disadvantage	116.80	118.32	1.01 (0.84–1.23)	46.06	48.12	1.04 (0.90–1.22)
	Average SE disadvantage	82.01	79.19	0.97 (0.77–1.22)	37.99	34.51	0.91 (0.76–1.09)
	High SE disadvantage	101.28	83.04	0.82 (0.66–1.02)	39.78	38.22	0.96 (0.80–1.16)
	Very high SE disadvantage	97.04	91.21	0.94 (0.75–1.17)	42	43.02	1.02 (0.83–1.26)

*Note:* IRR that are significant are highlighted in bold. *Population estimates used to calculate incidence rates are provided by IBIS Version 2021. **Early‐stage is defined as in situ or localized disease; late‐stage is defined as regional or distant disease.

Abbreviations: AIAN, American Indian or Alaskan Native; NH, Non‐Hispanic; NHPI, Native Hawaiian or Pacific Islander.

## 4 Discussion

The COVID‐19 pandemic impacted cancer screening and diagnostic services in ways that are still not fully understood. Reviewing the cancer data for the state of Utah revealed mixed results. Among cervical cancer cases in Utah, we observed a significant increase in advanced cervical cancer diagnoses during the pandemic compared with pre‐pandemic. We initially hypothesized that more time would be needed to observe increases in late‐stage cervical cancer diagnoses and that cervical cancer diagnoses would not change remarkably during the pandemic, but this did not turn out to be the case. The proportion of distant cervical cancer cases increased from 9.8% of all diagnosed cases in 2018–2019 to 24.6% during the pandemic (2020–2021). The overall incidence of cervical cancer was also higher during the pandemic period, despite the severe disruption to screening and diagnostic procedures at the start of the pandemic.

We hypothesize several possible explanations for this observation. First, there is some indication that late‐stage cervical cancer actually did begin increasing shortly after the pandemic onset. For example, Dellino et al. found evidence of increased incidence of later stage cervical cancer after the pandemic, which is likely attributable to the suspension of follow‐up [22]. A recent nationwide study also observed the proportion of late‐stage diagnoses for cervical cancer increased in 2020 and again in 2021, compared to 2017–2019 [23]. Second, our results could be explained by longer term changes in cervical cancer screening behaviors in Utah. According to Utah BRFSS data, Pap test screening rates among women in Utah have been steadily declining over the years, from 74.3% in 2010 to just 62.9% in 2020 [3]. This could indicate that the rise in distant‐stage cervical cancer diagnoses observed in this study may be a consequence of waning adherence to screening guidelines over time, not a specific impact of the COVID‐19 pandemic. This hypothesized reason for the observed increase in cervical cancer incidence is supported by a recently published study that assessed cervical cancer screening and diagnosis data in the United States between 2001 and 2018. The study demonstrated a steep increase in new cases of stage 4 cervical cancer over the study period, with the authors citing a decrease in screening trends as a likely cause [24]. Another possible explanation for the increase in late‐stage cervical cancer during the pandemic could be that because cervical cancer incidence is relatively low and the number of new cases in a given year in Utah is small, the fluctuations we observed between the pandemic and pre‐pandemic period are due to chance rather than attributable to differences in health care due to the COVID‐19 pandemic.

As for breast cancer, we observed a significant decrease in the incidence rate of local‐stage breast cancer and AJCC stage I breast cancer in the pandemic period compared to the pre‐pandemic period, as hypothesized. However, we saw no statistically significant change in the rates of in situ and overall breast cancer incidence between the same time periods, and no other AJCC stage showed statistically significant changes during the pandemic. There was a slight increase in later AJCC‐stage diagnoses in the pandemic, albeit not statistically significant. One possible hypothesis for why overall breast cancer incidence did not decline may be due to this slight shift in later stage diagnoses. Additionally, while national rates of breast cancer screening dropped during the pandemic, mammography adherence in Utah over the course of 2020 did not decrease significantly compared to prior years. According to Utah BRFSS data, in 2020 62.7% (95% CI 60.6%, 64.7%) of Utah women 40 years and older reported having received a mammogram within the past 2 years compared to 63.8% (95% CI 60.9%, 66.6%) in 2019 [2]. One major health system in Utah only reported a 4% decline in breast cancer screening during the pandemic compared to pre‐pandemic [25]. This refutes initial concerns that women in Utah may be more vulnerable to worsening breast cancer outcomes as a result of the pandemic decreasing Utah's mammography screening rates, which are low compared to the rest of the United States and place Utah among the bottom of the states in screening adherence. However, there is indication that breast cancer diagnoses did drop significantly in April 2020 in Utah and elsewhere in the U.S.; particularly early‐stage diagnoses [14]. Thus, it is important for us to continue monitoring trends in stage at diagnosis to fully understand the impact of the pandemic on trends in late‐stage diagnoses as well as outcomes such as mortality. As stage I disease survival is much better than late‐stage diagnosis [26], any delay in diagnosis could have long term consequences for survival.

Research has provided varying estimates for the impact of the COVID‐19 pandemic on breast and cervical cancer screening and cancer diagnoses in the United States. Multiple studies have demonstrated substantial drops in screening, particularly during the initial stay‐at‐home orders beginning in March 2020 [8, 27, 28, 29, 30]. Yet, one study using BRFSS data for the United States found that the prevalence of having received breast and cervical cancer screening in the past year only declined by 6% and 11%, respectively, between 2018 and 2020 [31]. However, such decreases were not observed in Utah. In Utah, the two largest healthcare organizations both paused nonurgent care such as cancer screenings for at least 6 weeks, starting 3/16/2020, and began rescheduling patients for early to mid‐May [32]. Thus, it is plausible that whereas screening was temporarily paused, the pause was relatively short and most affected patients were able to be screened later in the year after healthcare facilities opened back up. This is consistent with a nationwide study that showed that while breast cancer screening dropped sharply in March through May 2020 compared to the prior year, it had nearly completely recovered by July [8]. However, other research indicates that nationwide, cancer screening had not yet fully recovered compared to pre‐pandemic levels in 2021 [33]. Additionally, diagnostic mammograms and diagnoses dipped during this time and had not fully rebounded by 2022 [6]. Unfortunately, screening prevalence estimates derived from sources such as BRFSS data do not provide us with month‐by‐month estimates, making it difficult for us to fully understand population prevalence of screening in Utah in more fine‐grained detail throughout the course of 2020 and 2021. Nevertheless, numerous studies indicate that the COVID‐19 pandemic affected cancer screening, as well as diagnosis and treatment initiation for cervical cancer [7, 34] and breast cancer [28, 29, 35, 36]. Recent nationwide data suggest that while incidence of all cancers declined in 2020, for the most part, 2021 incidence was back up to expected rates [23].

Interestingly, we did not observe changes in breast and cervical cancer incidence or stage at diagnosis between the pandemic and pre‐pandemic periods among certain medically vulnerable and underserved populations, including those without health care coverage or living in high poverty areas. Incidence of late‐stage cervical cancer did increase among residents of higher socioeconomically disadvantaged areas, indicating some evidence of disproportionate impact on underserved populations. Additionally, we found that late‐stage breast cancer increased among Hispanic women during the pandemic period. This finding is particularly concerning as Hispanic or Latina women experience worse mortality outcomes and are more likely to be diagnosed with aggressive tumors than non‐Hispanic White women [37]. Thus, delays in diagnosis can have grave implications for their prognosis. Due to small numbers of cancers diagnosed in women who are a race other than White in Utah, we were unable to fully assess the impact of the pandemic on underserved racial groups, and consequently, we may be misrepresenting the true impact of the pandemic on these populations. For reference, the racial and ethnic minority population in the United States is approximately 41.1%, whereas in Utah it is just 23.3% [38]. Longer follow‐up and a larger study population are needed to fully evaluate the potential increased disparities in breast and cervical cancer due to the pandemic. One possible explanation for why we did not see as many disparities in late‐stage diagnosis as originally hypothesized may be that the pandemic did not impact health care access in Utah as much as in other areas of the country. Despite the downward trend in Pap test screening discussed earlier in this section, from 2018 to 2020, screening adherence in Utah did not differ significantly [13]. However, it is important to recognize that systemic barriers to health care that affect underserved populations persisted or worsened during the pandemic. Research indicates that in some cases, existing disparities in breast and cervical cancer screening were exacerbated by the pandemic, disproportionately affecting some segments of the population such as disabled individuals [39], Hispanic women [40], and the economically disadvantaged [41]. More research is needed to evaluate pandemic‐related screening patterns by social determinants of health in Utah.

A strength of this study is the use of population‐based data from a central cancer registry, which provides more generalizable results than studies that rely on data from a single health system. A limitation of this data source is that it does not include screening information for patients or details on the method of cancer detection, which would have allowed for better evaluation of the impact of screening reduction on stage at diagnosis and disparities. Our study is also limited by the short follow‐up period we were able to analyze after the onset of the COVID‐19 pandemic. More extensive analysis and longer follow‐up may be needed to assess the full extent to which the disruption caused by the COVID‐19 pandemic may have impacted breast and cervical cancer trends, such as later stage diagnoses and excess mortality. Additionally, the lack of diversity among Utah's population makes it difficult to analyze gaps in advanced cancer cases. When disaggregating our data by socioeconomic factors, many of our case counts were relatively small and therefore may not have had the statistical power to expose such a small effect. Future research should focus on the long‐term impact of the COVID‐19 pandemic on patient stage progression, as well as consider aggregating more years of data as they become available in order to investigate potential gaps in diagnostic and survival outcomes.

## 5 Conclusions

Incidence rate of local‐stage breast cancer in Utah declined significantly during COVID‐19 compared with pre‐COVID‐19; however, incidence rates among all other stages of breast cancer diagnoses remained stable. Conversely, we observed a significant increase in the incidence rates of distant stage cervical cancer and all invasive cervical cancers during the pandemic compared with pre‐pandemic. In considering the latency in breast and cervical cancer diagnosis, more time may be needed to realize the full impact of the COVID‐19 pandemic on breast and cervical cancer diagnoses in Utah.

## Author Contributions

**Michelle Mumper:** conceptualization (lead), formal analysis (lead), investigation (lead), methodology (lead), project administration (lead), validation (lead), visualization (lead), writing – original draft (lead), writing – review and editing (equal). **Leisha Nolen:** supervision (lead), writing – review and editing (equal). **Kimberly A. Herget:** conceptualization (equal), data curation (equal), methodology (equal), project administration (equal), writing – review and editing (equal). **Rachel R. Codden:** conceptualization (supporting), writing – review and editing (equal). **Marjorie E. Carter:** data curation (equal), writing – review and editing (equal). **Marie Nagata:** writing – review and editing (equal). **Morgan M. Millar:** conceptualization (equal), methodology (equal), project administration (equal), writing – original draft (equal), writing – review and editing (equal).

## Disclosure

IRB statement: IRB approval was not needed as the study was conducted by a public health entity using surveillance data collected under public health surveillance authority. This is in accordance with SOP 401A: NON‐HUMAN SUBJECT RESEARCH which states:

“*For the purposes of this policy, the following activities are not considered research*:

- *Public health surveillance activities, including the collection and testing of information or biospecimens, conducted*, *supported, requested, ordered, required, or authorized by a public health authority*.”

## Consent

Informed consent is not applicable, as this is a surveillance study using existing public health surveillance data.

## Conflicts of Interest

The authors declare no conflicts of interest.

## Data Availability

The data that support the findings of this study are available from the Utah Cancer Registry upon request after obtaining relevant regulatory approvals.
